# How Does a Company’s ESG Performance Affect the Issuance of an Audit Opinion? The Moderating Role of Auditor Experience

**DOI:** 10.3390/ijerph20053878

**Published:** 2023-02-22

**Authors:** Xin Wang, Xiayun Song, Mingyang Sun

**Affiliations:** 1 School of Business, Fuyang Normal University, Fuyang 236037, China; 2 Graduate School of Business, SEGI University, Petaling Jaya 47810, Malaysia; 3 School of Accounting, Zhejiang University of Finance and Economics, Hangzhou 310012, China; 4 School of Accounting, Tianjin University of Finance and Economics, Tianjin 300222, China

**Keywords:** ESG (environment, society, governance), auditor experience, audit opinion

## Abstract

Green economic development is a new growth point for China’s economy. The reduction in environmental pollution and the practice of social responsibility are strongly advocated by society. ESG (environment, society, governance) is a new concept considering how companies achieve sustainable development. Do auditors pay attention to corporate ESG when making opinion decisions? This paper examines how ESG performance affects audit opinion decisions. The results show the following: (1) The better the ESG performance is, the lower the probability of a modified audit opinion on the part of the auditor will be. (2) Consideration of the auditor experience indicates that auditors who lack experience rely more heavily on information about a corporate ESG performance to make their opinion decisions. (3) The mechanism test demonstrated that a sound ESG performance improves the quality of its financial reporting, which, in turn, reduces the probability of the auditor issuing a modified audit opinion. These conclusions remain robust after considering a number of tests, such as changing variable measures and endogeneity issues. This research expands the study of the economic consequences of ESG from an audit perspective, providing new evidence regarding the importance that corporate management places on ESG performance and how market intermediaries use ESG information.

## 1. Introduction

Sustainable development is a global concern. An increasing number of countries are introducing policies calling for the inclusion of ESG (environment, society and governance) factors in the capital market investment decision system and requiring listed companies to voluntarily or mandatorily ensure ESG information disclosure [1]. Capital markets have responded to corporate ESG disclosure. For example, companies with a good ESG performance have better stock market returns [2], and institutional investors have a more pronounced preference for ESG investments [3]. Additionally, on the company level, a sound ESG performance can improve financial performance [4,5] and reduce financing costs [6] and operational risks [7,8]. Since a company’s ESG performance can signal effects on itself and market stakeholders, do auditors, as key intermediaries in the capital markets, pay attention to corporate ESG performance information? Does a sound ESG performance, for example, reduce the probability that an auditor will issue a modified audit opinion (MAO)? After all, the impact of MAO on the capital market is relatively large, including an increased probability of delisting and other serious negative consequences [9].

In the past three decades, China has paid an abundance of environmental and social costs with its rapid economic growth. Although the per capita carbon emissions are not high, the total carbon emissions rank first in the world. Company production, relative to carbon emissions generated by living, is a significant cause of this result [10]. The ESG concept emphasizes the importance of environmental protection, the fulfillment of social responsibilities and the improvement of governance, being highly consistent with the new development concept advocated by China.

This paper examines the relationship between ESG performance and audit opinion using data of Chinese A-share-listed companies in Shanghai and Shenzhen from 2009 to 2020 and demonstrates that the ESG performance of companies is adversely related to the probability that auditors will issue an MAO. Auditor experience plays a significant moderating effect, while financial report quality plays a significant mediating role.

The contributions of this paper mainly concern two aspects. Firstly, it expands the study of the consequences of ESG performance from the audit opinion decision, revealing, in particular, how the auditor’s working experience moderates the audit opinion decision made by the auditor using the client’s ESG information, which is a major innovation relative to the existing studies. It also enriches the literature on auditor experience. Secondly, we clarify the mediating mechanism of ESG performance in regard to the audit opinion decision from the perspective of financial reporting quality, enriching the theoretical findings on the audit opinion decision from a new perspective. 

## 2. Literature Review

### 2.1. Research on the Economic Consequences of ESG Performance

On the role of ESG performance in corporate performance, firstly, one should note that many scholars have found that a sound ESG performance can improve financial performance [4,5,11]. However, using data from 104 multinational firms, Duque-Grisales and Aguilera-Caracuel (2021) found a significant negative correlation between ESG scores and financial performance [12]. Secondly, a sound ESG performance minimizes corporate risk. Better ESG disclosure can significantly reduce default risk [7] and credit risk [8]. Third, what is the reaction of the capital market to ESG information? ESG information disclosure can increase the value of the company’s market [13]. The disclosure of important ESG information can increase the amount of stock price information, so that the stock price can better reflect the company-level information [14]. Fourth, some scholars have explored the impact of ESG information on the cost of corporate finance. Due to the more efficient use of financial, human and social capital, ESG-performing firms are more competitive, have fewer systemic risks, and are expected to have lower financing costs [6]. However, some scholars have found different results, that is, a high volume of ESG data disclosed by companies or a high average cost of capital as a result [15,16].

On the effect of ESG performance on external stakeholders, firstly, one should note that for fund managers, the active portfolio management of ESG has potential advantages [17], and funds that invest in assets that conform to the philosophy of ESG perform better than funds that invest in lower ESG [18]. Moreover, the return volatility of the ESG portfolio is smaller than that of the benchmark market portfolio [19]. Secondly, from the auditor’s perspective, for clients with a poor ESG performance, the auditor must increase their audit efforts to reduce the risk of the client’s poor ESG reputation [20]. In contrast, auditors are more willing to reduce audit fees for companies with better ESG rating results [21]. Third, from the perspective of the rating agencies, the more ESG information is disclosed, the greater the divergence in the ESG ratings will be [22]. Different rating agencies have different priorities and standards. Regarding investors, they should not blindly compare the rating results but pay attention to their own investment strategies and select ESG rating data with a focus on their investment strategies [23]. In addition, Arvidsson and Dumay (2022) argue that what needs to be improved is ESG performance, rather than focusing on the quantity and quality of reporting. The cart should not be put before the horse, so that some environmental and social issues such as climate change can be mitigated [24].

### 2.2. Research on the Factors Influencing Audit Opinion Decisions

As far as the audit firm is concerned, clients paying more fees for non-audit services are more likely to receive a clean audit opinion, either because the consulting services helped the client to resolve the issue or because the auditor’s independence was compromised [25,26]. The incidence of audit firms offering lower-quality audits issuing going concern opinions to troubled companies is low [27]. Secondly, for the individual auditor, studies have shown that auditors with more experience in their work can inhibit management opportunistic behavior and prefer to issue modified audit opinions for high-risk clients [28], whereas the long tenure of the auditor [29] and the existence of social relationships between executives and the auditor [30,31] increase the probability of a clean audit opinion being issued.

The auditor’s opinion preferences vary between clients with different characteristics. Auditors are more likely to issue a going concern opinion following a ruling on a company on the verge of bankruptcy [32]. For important clients, auditors are more cautious and more likely to provide an MAO [33]. Conversely, auditors prefer to issue clean audit opinions for companies with a high degree of environmental disclosure [34] and better-quality internal controls [35]. In addition, the financial characteristics of the client can influence the auditor’s decision on a going concern opinion [36]. For clients experiencing financial difficulties, auditors are unlikely to issue an MAO when corporate management has greater control, which may be due to the fact that greater management power generates more purchases of audit opinions [37].

From the external perspective, negative news reports increase the auditor’s perception of the customer’s probability of bankruptcy, which, in turn, leads the auditor to issue an MAO [38]. A culture of confidentiality can affect the issuance of an audit opinion. If the client’s country has a stronger culture of confidentiality, the auditor will be more likely to issue an MAO [39]. Investor investment emotions [40] can promote audit opinion purchases, while effective monitoring can discourage them [41].

In summary, current research on the economic effects of ESG mostly includes the impacts on business operations and external stakeholders. The researcher contributions to the factors influencing audit opinion are mainly related to the audit party (audit firm and auditor), the audited client and the external circumstances. Among these factors, the characteristics of the auditee have always been a significant topic. Researchers have studied the impacts of the company’s equity structure, industry characteristics, financial characteristics or corporate governance on the audit opinion but have seldom discussed the correlation between the company’s overall environment, its social and governance practices and the audit opinion. Literature on the consequences of ESG from an audit opinion perspective is relatively scarce. From the perspective of the auditor’s experience, there has been even less research conducted on how the auditor uses the client’s ESG information to make audit opinion decisions. With the development of China’s capital market, the quality and requirements of corporate information disclosure have gradually increased, and the importance of audit opinions in the judgment or decision making processes of enterprises and investors has never been higher. Does ESG, as an important element of the non-financial information presented of a company, affect the audit opinion, and if so, how? Further research is needed on this issue. 

## 3. Hypothesis Development

### 3.1. Does ESG Performance Affect Audit Opinion Decisions?

Auditors are increasingly focusing on the non-financial information of companies when they conduct their engagements to express an audit opinion. The fulfillment of social responsibility by corporate managers will reduce surplus management [42] and eliminate the negative effects of irresponsible behavior. The quality of a firm’s environmental disclosure affects the cost of capital and market value [43], which are factors that auditors consider when issuing an opinion. Additionally, good corporate governance and social responsibility fulfillment can reduce the business risks and financial risks of the company, rendering the auditor’s risk perception level lower and thus encouraging them to be more willing to issue an unqualified opinion.

According to the theory of information asymmetry, auditors have a strong willingness to assess business risks out of self-interest, which can further influence the decisions they make. The company, as the audited party, actively discloses information to declare its good operation, so that the auditor’s decision will be more favorable for its development. ESG disclosures can, to some extent, reduce auditor information costs and information disadvantages [44], help auditors to better assess the current risks faced by firms and future growth expectations [45], and thus reduce auditor forecasting errors [46].

According to signaling theory, ESG performance can signal corporate risk and reduce auditor’s auditing risk. As China’s will to protect the environment becomes stronger and environmental laws and regulations become better, listed companies face more stringent environmental inspections. A good ESG performance means that the company has assumed a relatively high level of environmental and social responsibility and has a good internal corporate governance structure, which are important factors for the auditor to consider when issuing an audit opinion. A higher ESG score usually means that the company is more proactive in its environmental and social responsibilities, which helps to improve the auditor’s overall assessment of the company and thus reduces the probability of an MAO being issued. Hypothesis 1 is proposed as follows:

**H1:**  *The better the ESG performance of a company is, the less likely the auditor is to issue an MAO*.

### 3.2. Moderating Role of Auditor Experience in the Relationship between ESG Performance and Audit Opinion

Generally, auditors with extensive experience are highly capable of detecting material mis-statements in audited entity financial reports [47], reducing the probability of mis-statements in financial statements [48] and promoting efficient corporate investments [49] and audit efficiency [50]. Audit quality is higher when the auditor has more appropriate expertise [51] and audit experience [52]. Additionally, auditors who consecutively audit a company show a better financial reporting quality [53]. The audit risk of auditors with a long tenure and great experience is lower than the audit risk observed after changing auditors due to sufficient knowledge of the client [54]. However, for clients who pay their auditors unusually high compensation, a longer auditor tenure can reduce the audit quality [55].

Studies have found that auditor experience weakens the negative correlation between a trustworthy CFO and audit fees [56]. Thus, can auditor experience strengthen or weaken the influence of ESG on audit opinions? According to the analysis above, from the perspective of auditor experience, inexperienced auditors tend to rely more on external ESG rating information in the audit evidence collection process as far as ESG performance is concerned. Conversely, if the auditor is experienced, this competent auditor will rely more on audit expertise to issue audit opinions and rely less on ESG rating information. Therefore, this paper hypothesizes that auditor experience attenuates the impact of ESG on audit opinions. Hypothesis 2 is proposed as follows:

**H2:**  *Less experienced auditors rely on more ESG information to make audit opinion decisions than more experienced auditors*.

### 3.3. How Does ESG Performance Affect Audit Opinion Decisions? The Mediating Role of Financial Reporting Quality

Firstly, corporate ESG performance can improve the quality of financial reporting, thereby increasing the probability that the auditor will issue an unqualified opinion. Firms whitewash their statements to hide bad news that affect going concern, and a good ESG performance can improve financial performance [4] and stock market returns [2] and reduce corporate risk [7] and financing costs [6]. As a result, companies with a good ESG performance have high potential for sustainable development, weaker incentives for management surplus manipulation and higher-quality financial reporting.

Secondly, companies with good a ESG performance are usually large-scale companies receiving more attention from the outside world. Based on reputation theory, companies actively fulfill and disclose their environmental and social responsibilities and strive to improve their corporate governance to safeguard their industry reputation from damage. In cases of financial fraud, these companies will pay a more serious price compared to average companies due to their higher level of external attention. As a result, companies with a good ESG performance are more stringent in controlling the quality of financial reporting. In terms of environmental performance, disclosure of environmental information by companies can significantly affect the quality of financial reporting [57]. Regarding governance performance, good corporate governance improves the quality of financial reporting, which in turn promotes sound resource allocation decisions by management [58]. As for social responsibility performance, companies that are more socially responsible [59] and ethical [60] tend to exhibit a better financial reporting quality. Capital market regulators should use CSR information as a guide to achieve a better allocation of capital market resources [61]. In addition, the high financial reporting quality of a company indicates that its manipulable surplus is smaller, and true and complete financial information disclosure can reduce the complexity of the auditor’s work and lower the auditor’s perceived level of audit risk, so that the probability of the auditor issuing an unqualified opinion will be higher. Hypothesis 3 is proposed as follows:

**H3:**  *The better the ESG performance is, the lower the probability that the auditor will issue an MAO by improving the quality of financial reporting information.*

## 4. Research Design

### 4.1. Sample Selection and Data Sources

This paper examines the impact of corporate ESG performance on audit opinions, selecting a sample of Chinese listed companies in Shanghai and Shenzhen with A-shares from 2009 to 2020. The sample selection process is as follows. Firstly, samples of the financial and insurance categories are excluded due to industry specificity. Secondly, the samples with debt ratios greater than 1 or less than 0 are excluded. Third, the samples with missing variables are excluded, and finally, 28,645 firm–year observations are obtained. Fourth, the researcher carries out tailoring at the 1% level for all continuous variables so as to prevent the influences of extreme values on the outcomes. Data for the corporate ESG ratings are obtained from the Sino-Securities Index (SSI) database, data regarding auditor work experience and auditor tenure are obtained from audit reports, and data for other variables are obtained from the CSMAR database.

### 4.2. Variable Definition

#### 4.2.1. ESG Performance

The results of the SSI ESG ratings are selected as indicators to measure the ESG performance as the dependent variable. The rating database contains more than 3000 listed companies with A-shares in China, with a large sample size, and the conclusions obtained based on this sample analysis are highly convincing and reliable. The ESG rating results are divided into three major grades: A, B and C, and each major grade is subdivided into three minor grades. Therefore, the rating results can be divided into 9 grades from low to high. To facilitate the empirical analysis and quantitative processing in the following section of this article, in this paper, we assign the rating results of the nine grades of C~AAA from low to high, obtaining 1~9 points for quantification. (That is, when rated as AAA, ESG = 9; when rated as AA, ESG = 8; when rated as CC, ESG = 2; when rated as C, ESG = 1, etc.)

#### 4.2.2. Audit Opinion

In China, there are five types of audit opinions: unqualified opinion, unqualified with emphasis of matter, qualified, disclaimed and adverse opinion. Except for unqualified opinion, the other four types of audit opinions are included in the modified audit opinion (MAO). In order to convert audit opinions into measurable indicators for further quantification, the unqualified opinion is assigned a value of 0 and the MAO is assigned a value of 1 in this paper.

#### 4.2.3. Auditor Experience

The more companies an auditor audits, the more experience the auditor gains. In this paper, we use the natural logarithm of the total assets of all companies audited by the auditor in the client’s industry cumulatively to measure auditor experience. Since there are two signatory auditors in the corporate audit report, in this paper, we take the greater of the two signatory auditors’ experience indicators.

#### 4.2.4. Financial Reporting Quality

Many scholars use earnings management as a substitute for financial reporting quality (FRQ), but others believe that using a single indicator to measure FRQ is not reasonable and can lead to serious bias. Therefore, in this paper, we use the following three different models to calculate manipulability accruals.

(1) $$\frac{TA_{i,t}}{A_{i,t-1}}=\alpha_{1}\frac{1}{A_{i,t-1}}+\alpha_{2}\frac{\Delta SALES_{i,t}-\Delta AR_{i,t}}{A_{i,t-1}}+\alpha_{3}\frac{\Delta PPE_{i,t}}{A_{i,t-1}}+\varepsilon_{i,t}$$

In model (1), *TA* is the total accrual, which is equal to the gap that exists between operating profit and cash flow derived from the operating activities for the year; Δ*SALES* is the increment in sales revenue, Δ*AR* is the increment in the enterprise accounts receivable, Δ*PPE* is the increment in net fixed assets, and *A_i,t_*_−1_ is the firm’s total assets at the previous year end.

(2) $$\frac{TA_{i,t}}{A_{i,t-1}}=\beta_{1}\frac{1}{A_{i,t-1}}+\beta_{2}\frac{\Delta SALE_{i,t}}{A_{i,t-1}}+\beta_{3}\frac{\Delta PPE_{i,t}}{A_{i,t-1}}+\beta_{4}Roa_{i,t}+\lambda_{i,t}$$

In model (2), *Roa* is the firm’s return on assets.

(3) $$\frac{TA_{i,t}}{A_{i,t-1}}=\delta_{1}\frac{CFL_{i,t-1}}{A_{i,t-1}}+\delta_{2}\frac{CFL_{i,t}}{A_{i,t-1}}+\delta_{3}\frac{CFL_{i,t+1}}{A_{i,t-1}}+\delta_{4}\frac{\Delta SALES_{i,t}}{A_{i,t-1}}+\delta_{5}\frac{\Delta PPE_{i,t}}{A_{i,t-1}}+\eta_{i,t}$$

In model (3), *CFL* is the cash flow derived from operating activities.

Firstly, model (1)–(3) is regressed by industry and year, and the residuals are obtained, which are manipulable accruals. Secondly, the residuals are multiplied by −1 as an absolute value and normalized. Finally, the average of the three is taken as the FRQ, and the larger the FRQ is, the higher the financial reporting quality of the firm will be.

#### 4.2.5. Control Variables

With reference to the relevant studies [7,62,63], the following factors that may affect the audit opinion are selected as control variables in this paper. Regarding financial characteristics, we control for the enterprise size (Size), debt ratio (Debt), net cash flow from operating activities per share (CF), liquidity ratio (Liqt), return on assets (Roa) and growth rate of operating income (Growth). In terms of corporate governance, we control whether the CEO concurrently serves as the COB (CC) and the shareholding ratio of the top shareholder (Top). Externally, we control the level of marketization (Market). In addition to this, the model controls for the firm’s industry (Industry) and year (Year) as fixed effects in order to exclude interference between different years and industries. The variables are defined in Table 1.

### 4.3. Estimation Model

To test the impact of corporate ESG performance on audit opinion, we draw on Benlemlih and Bitar’s (2018) study, constructing a logit regression model (4) to verify H1 [64].

*MAO* = *α*_0_ + *α*_1_*ESG* + ∑ *Controls* + ∑*Year* + ∑*Ind* + *ε* (4)

Controls in the model denote the control variables. The model also controls for year effects (Year) and industry effects (Ind), with *ε* as the random error term in the model. A significant coefficient *α*_1_ in the model implies that there is a significant effect of corporate ESG performance on audit opinion.

Based on model (4), model (5) adds auditor experience (Exper) and the cross-multiplier term of ESG and auditor experience (ESG × Exper) to examine the moderating effects of auditor experience on ESG and audit opinion, so as to test Hypothesis 2.

*MAO* = *α*_0_ + *α*_1_*ESG* + *α*_2_*Exper* + *α*_3_*ESG* × *Exper* + ∑ *Controls* + ∑*Year* + ∑*Ind* + *ε* (5)

The following models (6) and (7) are developed to test how ESG performance reduces the probability of auditors issuing an MAO by improving FRQ, where FRQ is the mediating variable, and the coefficients *β*_1_ and *δ*_2_ are tested in turn. If *β*_1_ is significantly positive, this means that the ESG performance can improve the FRQ. If *δ*_2_ is significantly negative, this means that a good financial reporting quality can reduce the probability of auditors issuing an MAO, and the Hypothesis H3 is confirmed.

*FRQ* = *β*_0_ + *β*_1_*ESG* + ∑ *Controls* + ∑*Year* + ∑*Ind* + *ε* (6)

*MAO* = *δ*_0_ + *δ*_1_*ESG* + *δ*_2_*FRQ* + ∑ *Controls* + ∑*Year* + ∑*Ind* + *ε* (7)

## 5. Results

### 5.1. Descriptive Statistics and Correlation Results

Table 2 below shows the descriptive statistics of all the variables. The results show that the mean and standard deviation of MAO are 0.038 and 0.191, respectively, showing that an average of 3.8% of the companies listed with A-shares in China from 2009 to 2020 were issued with modified audit opinions. The mean and standard deviation of corporate ESG ratings (ESG) are 6.462 and 1.126, respectively, with a minimum value of 1 and a maximum value of 9, suggesting that the overall rating results of the sampled companies are good but vary widely. The mean value of auditor experience (Exper) is 23.924, and the standard deviation is 1.439. The minimum value of the financial reporting quality (FRQ) is −1.513, and the maximum value is 0.396, which indicates that there are large differences in financial reporting quality between companies. The distribution of the corresponding values of the other control variables is basically within a reasonable range.

The correlation tests of the independent and dependent variables are shown in Table 3, and MAO is significantly and negatively correlated with ESG. The Spearman and Pearson correlation coefficients of both the independent and dependent variables are below 0.5. Empirically, coefficients less than 0.7 indicate that the model is basically free of multicollinearity problems and does not affect our results.

### 5.2. Regression Results

#### 5.2.1. Impact of Corporate ESG Performance on Audit Opinion (H1)

Column (1) of Table 4 shows the results of the univariate regression of the audit opinion and ESG performance without the inclusion of control variables. Column (2) shows the results of the regression with the inclusion of control variables. They both show that the coefficient of ESG is significantly negative at the 1% level. This indicates that the better the ESG performance of the firm is, the lower the probability that the auditor will issue an MAO when making audit decisions will be. This may be due to the fact that the better the degree of corporate governance and disclosure of the firm are, the lower the information risk and operational risks influencing the auditor to lower the audit risk will be, thus reducing the likelihood of the auditor issuing a modified audit opinion. This is consistent with the expectation of H1.

#### 5.2.2. The Moderating Role of Auditor Experience (H2)

The regression results of model (5) are reported in column (3) of Table 4. The results show that the interaction term between ESG and auditor experience (ESG × Exper) is significantly positive. This indicates that auditor experience attenuates the effect of ESG on audit opinion, which means that the more experienced the auditor and the less their need to rely too much on ESG information are, the less the effect of ESG rating on audit opinion will be. This results support H2.

#### 5.2.3. The Mediating Role of Financial Reporting Quality (H3)

For a positive ESG performance to play a role in audit opinions, it must also rely on specific paths. The mechanism by which ESG performance affects audit opinion was illustrated in terms of FRQ in the previous section, and this section examines the mechanism that works to produce this effect. Referring to Wen and Ye (2014) [65], first, the regression results of ESG and its effect on audit opinion are tested, as described in column (2) of Table 4. Second, the results of ESG on FRQ are examined in column (4) of Table 4, and the ESG coefficient is 0.017, which is significantly positive at the 1% level, indicating that a better ESG performance can improve FRQ. Third, the mediating variables are added to the baseline regression in the first step, and the results in column (5) of Table 4 demonstrate that the coefficient of FRQ is significantly negative, indicating that the higher the quality of financial reporting is, the lower the probability of the auditor issuing an MAO will be. The data test is consistent with the expectation of H3, indicating that a good ESG performance reduces the probability of the auditor issuing an MAO by improving the FRQ.

### 5.3. Robustness Tests

#### 5.3.1. Changing the Measurement of Corporate ESG Performance

In the regression analysis above, the ESG performance is based on the ESG rating results, and the nine grades are assigned from low to high as 1–9. To check the robustness of the results, the rating results are reclassified, and C, CC and CCC are assigned as 1; B, BB and BBB are assigned as 2; and A, AA and AAA are assigned as 3. The results of model (4) after replacing the ESG performance measure are shown in column (1) of Table 5, the results of model (5) are shown in column (2) of Table 5, and the results of models (6) and (7) are shown in columns (3) and (4) of Table 5, and the results are still significant.

#### 5.3.2. Changing the Measurement of Audit Opinions

In the regression analysis above, the audit opinion is assigned a value of 0 for an unqualified opinion and 1 for a modified audit opinion. To test the robustness of the results, the audit opinion is reclassified as Mao4. The unqualified opinion is assigned a value of 1; the unqualified opinion with emphasis of matter is assigned a value of 2; the qualified audit opinion is assigned a value of 3; and the adverse or disclaimed opinion is assigned a value of 4. The higher the value of Mao4 is, the worse the audit opinion issued by the auditor will be. The results of models (4), (5), (6) and (7) after replacing the measurement of audit opinion are shown in columns (1), (2), (3) and (4) in Table 6, and the regression results are still significant.

#### 5.3.3. Changing the Measurement of Auditor Experience

For the measurement of auditor experience, as shown above, the auditor’s cumulative client assets audited in the client’s industry are used. To test the robustness of the results, auditor tenure is used here as a proxy for auditor experience. The longer the auditor tenure is, the greater the audit experience is. The tenures of the two signatory auditors are averaged and expressed as Exper_mean. The results with Exper_mean integrated into in model (5) are shown in column (5) of Table 6 and remain robust.

### 5.4. Endogeneity Bias

The variables selected for the paper may have potential endogeneity issues. For example, the ESG scores are high, because the firms themselves have adequate disclosure and a better internal control environment, which, in turn, means that the auditors are less likely to issue an MAO. Considering the sample selection bias and verifying the causal relationship between ESG performance and audit opinion, the following methods are used to check this paper: the Heckman correction method, propensity score matching method (PSM), instrumental variables method and the addition of control variables, respectively.

#### 5.4.1. Heckman Correction Method

Since ESG information is voluntarily disclosed, there are firms that do not disclose this information and, therefore, do not appear in our research sample. To address this problem, the Heckman correction method is used in this paper. First, a dummy variable for ESG performance, ESG_dum, is generated and assigned a value of 1 if it is above the sample median or 0 if it is below the sample median. A probit regression is conducted with ESG_dum as the explanatory variable, and the results are shown in column (1) of Table 7. Second, the inverse Mills ration (IMR) is calculated, and the IMR is significant, indicating the existence of sample selection bias in the model. With the incorporation of IMR as a control variable into the regression model, the results are shown in column (2) of Table 7. The coefficient of the explanatory variable, ESG, is significant, indicating that the sample selection bias is corrected using the Heckman correction method.

#### 5.4.2. Propensity Score Match

To alleviate sample selection bias, the PSM estimate is used in this paper for sample pairwise tests. First, the median of ESG is used as a boundary to classify the sample into two groups being greater than and less than the median. Second, the nearest neighbor matching method with 1:1 non-release sampling is used to construct the experimental and control group samples using the control variables selected in the paper as the matching criteria. Finally, the unmatched sample observations are removed, and the matched samples ae regressed. The results are still significant, as shown in column (3) in Table 7.

#### 5.4.3. Adding Control Variables

To mitigate the model bias caused by the omitted variables, the following control variables are added in this paper: whether or not the firm is in loss for the year (Loss), the book-to-market ratio (BM) and the management fee rate (MF). The results of model (4) are shown in column (4) of Table 7, and the results are still significant.

#### 5.4.4. Instrumental Variable Method

In this paper, the industry ESG mean (ESG_mean) of the company is selected as the instrumental variable. First, the two-stage least squares (2SLS) method is used to regress the instrumental variable (ESG_mean) with the ESG performance, as shown in column (1) of Table 8, and the coefficient is significantly positive, indicating that the instrumental variable is valid. Second, we regress the ESG (fitted) on MAO, and the results are shown in column (2) of Table 8, where the ESG performance is significantly negative at the 1% level with a modified audit opinion, which is consistent with the previous results.

## 6. Conclusions

Non-financial information is an important consideration that influences the auditor’s opinion decision. Companies seek to obtain unqualified audit opinions due to the adverse economic consequences of MAO. However, previous literature has provided less research on how ESG performance enhances the result of the audit opinion.

In this paper, we empirically examined the relationship between ESG performance and audit opinion using a sample of A-share-listed companies in Shanghai and Shenzhen from 2009 to 2020. It was found that the ESG performance of the companies is significantly and negatively correlated with MAO, verifying H1. Experienced auditors rely more on professional judgment, while inexperienced auditors rely more on ESG rating content when performing audit work. Therefore, auditors’ experience attenuates the effect of ESG on audit opinion, verifying H2. We also explored the channel mechanisms through which ESG performance influences audit opinions. In the study, we found that ESG performance can significantly enhance the quality of financial reporting, which, in turn, influences the auditor’s decision, verifying H3. The results remained robust after replacing the measurement of the main variables. To alleviate the endogeneity issue, the following methods were tested: the Heckman correction method, PSM, addition of control variables and instrumental variables methods. The results remained significant.

Our study makes a number of contributions to the literature. First, we expand the research on the economic consequences of ESG performance. Second, we enrich the literature on auditor experience by providing empirical evidence regarding the impact of auditor experience in moderating ESG information on auditor decision making. Third, our study contributes to the literature on the factors that influence auditor decision making.

The research has some practical implications. First, research on the economic consequences of ESG provides empirical evidence that can be used to guide firms to consciously improve their information disclosure. To obtain better auditor feedback, companies must embrace ESG concepts in all aspects of their operations and management and focus on environmental, social and governance synergies. Second, by promoting the sustainable development of enterprises, the green and sustainable development of China’s economy can be achieved. Third, factors influencing audit opinion are studied to provide a reference for the auditor intending to issue an opinion. At the same time, auditors should enrich their professional experience and not rely excessively on ESG information to avoid misjudgment in their audit opinions. Fourth, investors should concentrate on the ESG performance of companies before making investment decisions. Companies with a better ESG performance are more likely to receive an unqualified opinion, which can establish a good social reputation for the company, so that it is expected to yield better sustainable returns.

Since ESG information affects the auditor’s opinion decision, does it also affect other behavioral decisions of the auditor? More exploration can be conducted here to enrich the study of ESG information in regard to the auditor’s behavior. Due to the availability of data, the scope of this study is currently limited to China. Future studies could be conducted on data from other countries or regions so as to conduct additional research for cross-country comparative analysis.

## Figures and Tables

**Table 1:** Definitions of the variables.

Variable	Symbol	Definition
Modified Audit Opinion	MAO	A modified audit opinion obtained in the current year takes the value of 1; otherwise it takes the value of 0.
ESG Performance	ESG	Score assigned according to ESG rating results.
Auditor Experience	Exper	Auditor’s experience is measured by the logarithm of the auditor’s cumulative audited client assets in the client’s industry. This paper selects the greater of the two signatory auditors’ experiences.
Financial Reporting Quality	FRQ	According to the Formulas (1)–(3) calculation obtained.
Company Size	Size	Natural logarithm of total assets.
Debt ratio	Debt	The ratio of total debt to total assets.
Cash flow	CF	Net cash flow from operating activities per share.
Liquidity Ratio	Liqt	Liquid asset/Liquid debt.
Return on Assets	Roa	Net income/Total assets.
Growth	Growth	Growth rate of operating income.
Whether the CEO concurrently serves as COB	CC	The value of CEO and COB as one is 1; otherwise the value is 0.
Top shareholder holdings	Top	Largest shareholder shares as a percentage of total shares.
Degree of marketization	Market	Marketization Index of China’s Provinces (2019).
Year	Year	Yearly dummy variable.
Industry	Ind	Industry dummy variable.

**Table 2:** Descriptive statistics.

Variable	Obs	Mean	SD	P25	Median	P75	Min	Max
MAO	28,645	0.038	0.190	0.000	0.000	0.000	0.000	1.000
ESG	28,645	6.462	1.127	6.000	6.000	7.000	1.000	9.000
Exper	28,645	23.924	1.439	22.947	23.962	24.897	20.534	27.487
FRQ	28,645	0.056	0.350	−0.045	0.163	0.286	−1.513	0.396
Size	28,645	22.154	1.293	21.226	21.986	22.886	19.677	26.120
Debt	28,645	0.437	0.208	0.270	0.431	0.593	0.055	0.904
CF	28,645	0.046	0.071	0.007	0.045	0.087	−0.172	0.245
Liqt	28,645	2.356	2.409	1.104	1.607	2.579	0.296	15.851
Roa	28,645	0.035	0.063	0.013	0.035	0.065	−0.275	0.196
Growth	28,645	0.178	0.483	−0.033	0.101	0.262	−0.594	3.317
CC	28,645	0.266	0.442	0.000	0.000	1.000	0.000	1.000
Top	28,645	0.344	0.149	0.227	0.321	0.444	0.086	0.743
Market	28,645	8.809	7.498	3.000	6.000	13.000	1.000	29.000
Loss	28,645	0.104	0.306	0.000	0.000	0.000	0.000	1.000
BM	28,645	0.451	0.320	0.226	0.370	0.580	0.038	1.705
MF	28,645	0.094	0.081	0.045	0.074	0.115	0.009	0.526

**Table 3:** Correlation matrix of the independent and dependent variables.

Variable	MAO	ESG
MAO	1	−0.1538 ***
ESG	−0.1794 ***	1

Note: the upper and lower corners are Spearman and Pearson correlation coefficients, respectively, *** *p* < 0.01.

**Table 4:** Regression results.

Variable	(1) MAO	(2) MAO	(3) MAO	(4) FRQ	(5) MAO
ESG	−0.767 ***	−0.521 ***	−0.608 ***	0.017 ***	−0.515 ***
	(−27.702)	(−15.731)	(−14.245)	(5.391)	(−15.657)
Exper			−0.999 ***		
			(−2.692)		
ESG × Exper			0.193 ***		
			(3.057)		
FRQ					−0.257 ***
					(−5.735)
Size		−0.360 ***	−0.388 ***	0.026 ***	−0.355 ***
		(−9.183)	(−9.225)	(8.264)	(−9.086)
Debt		3.489 ***	3.476 ***	−0.183 ***	3.448 ***
		(13.641)	(13.559)	(−8.686)	(13.424)
CF		−1.605 ***	−1.592 ***	0.097	−1.701 ***
		(−2.934)	(−2.900)	(1.186)	(−3.373)
Liqt		0.041 *	0.040	−0.009 ***	0.034
		(1.711)	(1.626)	(−5.740)	(1.371)
Roa		−7.923 ***	−7.948 ***	0.885 ***	−7.346 ***
		(−18.017)	(−18.019)	(9.919)	(−16.042)
Growth		−0.027	−0.025	−0.226 ***	−0.097
		(−0.297)	(−0.278)	(−11.199)	(−1.036)
CC		−0.062	−0.057	−0.014 **	−0.066
		(−0.770)	(−0.698)	(−2.170)	(−0.815)
Top		−1.881 ***	−1.881 ***	−0.057 ***	−1.888 ***
		(−6.541)	(−6.509)	(−2.804)	(−6.575)
Market		0.014 ***	0.014 ***	−0.001	0.013 ***
		(2.921)	(2.959)	(−1.273)	(2.849)
_cons	1.367 ***	6.375 ***	7.438 ***	−0.615 ***	6.279 ***
	(8.644)	(7.439)	(7.945)	(−9.585)	(7.339)
Year/Ind	No	Yes	Yes	Yes	Yes
Pseudo-r^2^/r^2^_a	0.0945	0.2613	0.2626	0.104	0.2638
N	28,645	28,645	28,645	28,645	28,645

Note: T-values in parentheses, *** *p* < 0.01, ** *p* < 0.05, * *p* < 0.1.

**Table 5:** Changing the measurement of ESG.

Variable	(1) MAO	(2) MAO	(3) FRQ	(4) MAO
ESG_New	−1.028 ***	−1.270 ***	0.017 ***	−1.019 ***
	(−11.798)	(−10.437)	(2.935)	(−11.727)
Exper		−1.000 ***		
		(−2.756)		
ESG_New × Exper		0.503 ***		
		(3.004)		
FRQ				−0.279 ***
				(−5.908)
Size	−0.386 ***	−0.409 ***	0.030 ***	−0.380 ***
	(−9.937)	(−9.760)	(10.078)	(−9.801)
Debt	3.640 ***	3.621 ***	−0.189 ***	3.597 ***
	(14.089)	(13.975)	(−8.936)	(13.881)
CF	−1.642 ***	−1.640 ***	0.098	−1.737 ***
	(−2.972)	(−2.962)	(1.201)	(−3.427)
Liqt	0.043 *	0.042 *	−0.009 ***	0.036
	(1.811)	(1.737)	(−5.736)	(1.454)
Roa	−8.157 ***	−8.175 ***	0.906 ***	−7.533 ***
	(−18.586)	(−18.582)	(10.181)	(−16.431)
Growth	−0.035	−0.033	−0.227 ***	−0.108
	(−0.379)	(−0.352)	(−11.224)	(−1.126)
CC	−0.055	−0.050	−0.015 **	−0.059
	(−0.682)	(−0.617)	(−2.304)	(−0.730)
Top	−2.021 ***	−2.023 ***	−0.055 ***	−2.025 ***
	(−7.055)	(−7.036)	(−2.699)	(−7.073)
Market	0.013 ***	0.013 ***	−0.001	0.013 ***
	(2.802)	(2.807)	(−1.340)	(2.746)
_cons	5.910 ***	6.896 ***	−0.622 ***	5.807 ***
	(7.080)	(7.422)	(−9.763)	(6.964)
Year/Ind	Yes	Yes	Yes	Yes
Pseudo-r^2^/r^2^_a	0.2492	0.2504	0.103	0.2521
N	28,645	28,645	28,645	28,645

Note: T-values in parentheses, *** *p* < 0.01, ** *p* < 0.05, * *p* < 0.1.

**Table 6:** Changing the measurement of audit opinion and auditor experience.

Replacement Indicator	Audit Opinion				Auditor Experience
Variable	(1) Mao4	(2) Mao4	(3) FRQ	(4) Mao4	(5) MAO
ESG	−0.036 ***	−0.049 ***	0.017 ***	−0.035 ***	−0.582 ***
	(−14.750)	(−12.812)	(5.391)	(−14.484)	(−13.178)
Exper		−0.172 ***			
		(−5.308)			
ESG × Exper		0.027 ***			
		(5.570)			
FRQ				−0.047 ***	
				(−7.664)	
Exper_mean					−0.829 **
					(−2.281)
ESG × Exper_mean					0.140 **
					(2.252)
Size	−0.010 ***	−0.011 ***	0.026 ***	−0.008 ***	−0.363 ***
	(−4.629)	(−5.034)	(8.264)	(−4.039)	(−9.211)
Debt	0.166 ***	0.165 ***	−0.183 ***	0.158 ***	3.492 ***
	(8.303)	(8.291)	(−8.686)	(7.906)	(13.601)
CF	0.114 ***	0.117 ***	0.097	0.118 ***	−1.604 ***
	(3.293)	(3.390)	(1.186)	(3.432)	(−2.931)
Liqt	0.006 ***	0.005 ***	−0.009 ***	0.005 ***	0.041 *
	(5.915)	(5.811)	(−5.740)	(5.506)	(1.674)
Roa	−1.194 ***	−1.183 ***	0.885 ***	−1.152 ***	−7.849 ***
	(−15.886)	(−15.774)	(9.919)	(−15.676)	(−17.975)
Growth	−0.004	−0.004	−0.226 ***	−0.015 ***	−0.029
	(−0.899)	(−0.895)	(−11.199)	(−2.862)	(−0.322)
CC	−0.002	−0.001	−0.014 **	−0.002	−0.062
	(−0.426)	(−0.269)	(−2.170)	(−0.583)	(−0.770)
Top	−0.064 ***	−0.063 ***	−0.057 ***	−0.066 ***	−1.862 ***
	(−6.026)	(−5.929)	(−2.804)	(−6.286)	(−6.443)
Market	0.000	0.000	−0.001	0.000	0.014 ***
	(0.350)	(0.411)	(−1.273)	(0.254)	(2.983)
_cons	1.502 ***	1.625 ***	−0.615 ***	1.473 ***	6.733 ***
	(33.459)	(29.141)	(−9.585)	(32.779)	(7.745)
Year/Ind	Yes	Yes	Yes	Yes	Yes
Pseudo-r^2^/r^2^_a	0.117	0.119	0.104	0.122	0.2619
N	28,645	28,645	28,645	28,645	28,645

Note: T-values in parentheses, *** *p* < 0.01, ** *p* < 0.05, * *p* < 0.1.

**Table 7:** Heckman correction method/PSM/addition of control variables.

Variable	(1) ESG_dum	(2) MAO	(3) MAO	(4) MAO
ESG		−0.519 ***	−0.513 ***	−0.504 ***
		(−15.854)	(−12.770)	(−15.185)
IMR		−2.678 ***		
		(−4.369)		
Size	0.227 ***	−0.605 ***	−0.372 ***	−0.346 ***
	(21.289)	(−9.033)	(−7.618)	(−6.694)
Debt	−0.725 ***	4.326 ***	3.388 ***	3.845 ***
	(−9.697)	(13.810)	(12.191)	(14.805)
CF	0.476 ***	−2.077 ***	−1.491 **	−0.592
	(2.938)	(−3.718)	(−2.308)	(−1.037)
Liqt	0.005	0.043 *	0.028	0.025
	(0.865)	(1.808)	(0.890)	(1.134)
ROA	2.698 ***	−11.460 ***	−6.877 ***	−4.278 ***
	(16.072)	(−12.158)	(−14.788)	(−7.028)
Growth	−0.068 ***	0.062	−0.001	0.160 **
	(−3.208)	(0.674)	(−0.013)	(2.071)
CC	−0.061 ***	0.003	−0.150	−0.052
	(−2.652)	(0.033)	(−1.607)	(−0.630)
Top	0.625 ***	−2.473 ***	−1.799 ***	−1.460 ***
	(8.119)	(−7.586)	(−4.659)	(−5.024)
Market	−0.001	0.014 ***	0.007	0.004
	(−0.986)	(3.008)	(1.249)	(0.786)
Loss				0.781 ***
				(6.707)
BM				0.812 ***
				(4.654)
MF				4.331 ***
				(11.865)
ATT			0.0911 ***	
			(−5.52)	
_cons	−2.853 ***	11.642 ***	7.008 ***	4.793 ***
	(−11.663)	(7.935)	(6.361)	(4.443)
Year/Ind	Yes	Yes	Yes	Yes
r^2^_a	0.1352	0.2637	0.035	0.2884
N	28,645	28,645	7090	28,645

Note: T-values in parentheses, *** *p* < 0.01, ** *p* < 0.05, * *p* < 0.1.

**Table 8:** Instrumental variable method.

Variable	(1) ESG	(2) MAO
ESG_mean	0.859 ***	
	(48.14)	
ESG(fitted)		−0.016 ***
		(−4.31)
Size	0.293 ***	−0.012 ***
	(54.03)	(−7.61)
Debt	−0.463 ***	0.125 ***
	(−10.87)	(16.31)
CF	0.157 *	0.086 ***
	(1.73)	(5.28)
Liqt	0.005	0.004 ***
	(1.55)	(6.15)
ROA	2.257 ***	−0.703 ***
	(20.53)	(−32.24)
Growth	−0.111 ***	−0.000
	(−8.85)	(−0.14)
CC	−0.094 ***	0.000
	(−6.88)	(0.14)
Top	0.188 ***	−0.052 ***
	(4.57)	(−6.93)
Market	−0.003 ***	0.000 ***
	(−3.83)	(2.83)
Constant	−5.467 ***	0.371 ***
	(−35.78)	(18.08)
Observations	28,645	28,645
r^2^_a	0.218	0.109

Note: T-values in parentheses, *** *p* < 0.01, * *p* < 0.1.

## Data Availability

Not applicable.
